# Starling forces drive intracranial water exchange during normal and pathological states

**DOI:** 10.3325/cmj.2017.58.384

**Published:** 2017-12

**Authors:** Andreas A. Linninger, Colin Xu, Kevin Tangen, Grant Hartung

**Affiliations:** 1 Laboratory for Product and Process Design, Department of Bioengineering, University of Illinois at Chicago, Chicago, Illinois, USA; 2 Department of Neurosurgery, University of Illinois at Chicago, Chicago, Illinois, USA

## Abstract

**Aim:**

To quantify the exchange of water between cerebral compartments, specifically blood, tissue, perivascular pathways, and cerebrospinal fluid-filled spaces, on the basis of experimental data and to propose a dynamic global model of water flux through the entire brain to elucidate functionally relevant fluid exchange phenomena.

**Methods:**

The mechanistic computer model to predict brain water shifts is discretized by cerebral compartments into nodes. Water and species flux is calculated between these nodes across a network of arcs driven by Hagen-Poiseuille flow (blood), Darcy flow (interstitial fluid transport), and Starling’s Law (transmembrane fluid exchange). Compartment compliance is accounted for using a pressure-volume relationship to enforce the Monro-Kellie doctrine. This nonlinear system of differential equations is solved implicitly using MATLAB software.

**Results:**

The model predictions of intraventricular osmotic injection caused a pressure rise from 10 to 22 mmHg, followed by a taper to 14 mmHg over 100 minutes. The computational results are compared to experimental data with R^2^ = 0.929. Moreover, simulated osmotic therapy of systemic (blood) injection reduced intracranial pressure from 25 to 10 mmHg. The modeled volume and intracranial pressure changes following cerebral edema agree with experimental trends observed in animal models with R^2^ = 0.997.

**Conclusion:**

The model successfully predicted time course and the efficacy of osmotic therapy for clearing cerebral edema. Furthermore, the mathematical model implicated the perivascular pathways as a possible conduit for water and solute exchange. This was a first step to quantify fluid exchange throughout the brain.

The classical view of brain fluid circulation is that the choroid plexus actively secretes cerebrospinal fluid (CSF), which unidirectionally circulates through the ventricular spaces before being absorbed into the veins of the superior sagittal sinus via arachnoid granulations lining the subarachnoid space (SAS) (1,2). However, many experimental studies brought to question this classic theory of CSF circulation (3-8; Supplementary material 1(supplementary material 1)). A novel CSF circulation hypothesis proposed by Bulat et al (9-13) suggests that hydrostatic and osmotic pressure gradients induce water filtration and reabsorption across cerebral capillaries throughout the brain. Osmotic pressure gradients have been shown to be a driving force for water (14-19) and solute exchange (19); nascent fluid production in response to osmolar challenge (Supplementary material 2 (supplementary material 2)). According to the Bulat-Klarica-Orešković hypothesis (9-11,13,20), water is first filtered across the blood-brain barrier (BBB), but then reabsorbed into more distal capillaries or venules driven by a combination of hydrostatic and osmotic pressure gradients. Furthermore, mounting evidence from tracer (1,2,21-23), microscopy (24-26), and neuroimaging (27-30) studies suggest the existence of a preferential pathway for water and solutes within an annular region surrounding cerebral blood vessels known as the perivascular space (PVS). Both the morphology and the transport mechanisms active in the PVS are still the subject of discussion (2,24,31). Radioactive tracers injected into the SAS are rapidly dispersed into the interstitial fluid (ISF) space along penetrating arterioles (1,2,21). The fast rate by which radioactive tracers penetrate into the brain parenchyma suggests that solute transport is accelerated by convective mechanisms along the PVS (32).

In addition to the significant impact on CSF production, osmotic pressure gradients have also been reported to alter ventricular volume and intracranial pressure (ICP). Chronic injections of hyperosmotic fluid caused ventricular enlargement consistent with a reversible hydrocephalus-like state (18,33,34). Osmotic forces do affect ICP, yet ventricular enlargement induced by osmolarity challenges have yet to be reproduced by additional studies. Other data suggest a link between osmotic (35-38) and hydrostatic (36,39) pressure imbalances and cerebral edema. Vasogenic edema compromises BBB permeability, which allows proteins and ions to leak into the extracellular space (ECS) increasing the tissue osmotic pressure resulting in expansion of the ECS and increase in ICP. The most common treatment for cerebral edema is osmotic therapy, which consists of intravenous (IV) injection of mannitol or hypertonic saline. The theoretical method of action for osmotic therapy is that IV injected osmotic agents circulating in the blood stream raise plasma tonicity (Figure 1). When the blood reaches the brain, the osmotic gradient between plasma and the ECS reduces ICP by driving water from the parenchyma into the bloodstream.

**Figure 1:**
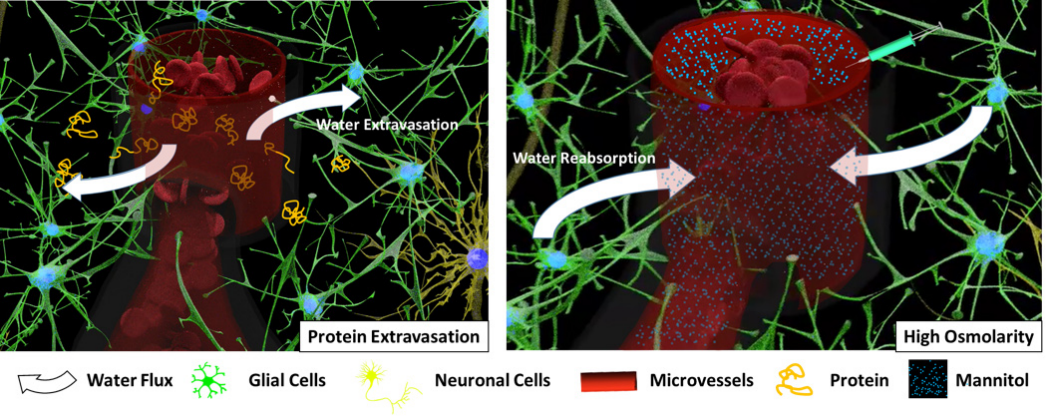
Illustration of cerebral edema and its clearance by osmotic therapy. Vasogenic edema occurs when the permeability of the blood brain barrier to water and solutes is increased due to mechanical trauma or endothelial cell disintegration. Blood proteins, ions, and water flood into the extracellular space increasing extracellular space (ECS) volume thus potentially causing intracranial pressure elevation. Protein from dying cells also augment ECS osmolarity, which in turn promotes water extravasation. On the other hand, osmotic therapy increases blood tonicity, which facilitates reabsorption of interstitial fluid into the blood, counteracting intracranial pressure rise.

Despite a wealth of experimental evidence, the role of physiological mechanisms driving CSF production, reabsorption, and hydrostatic pressures inside the brain are still topics of discussion (11,40). Much less quantitative knowledge is available for predicting water shifts across the BBB as a function of osmotic challenges. There is agreement that osmotic pressure gradients between blood, CSF, and ECS mediate water and solute exchange (9,14-19). Several computational models of intracranial dynamics simulate volume exchange and pressure interaction between the cerebral vasculature, CSF, and brain tissue (41-44). However, none of these studies account for the effect of osmotic pressure gradients on CSF production or intracranial water shifts. Most previous mathematical models also omit relevant structures, such as the cerebral microcirculation or PVS. Models that do include the PVS attribute water flux only to hydrostatic pressure gradients without accounting for contributions due to osmotic pressure (31).

The primary aim of this work was to establish a geometrically simplified, but mechanistically consistent computational framework to correlate both hydrostatic and osmotic driving forces with brain water shifts in normal and pathological conditions. It enabled the prediction of osmotic pressure and blood and CSF flow, in addition to transport through the porous brain. Model predictions were validated against the known body of experimental data and expected to elucidate the timing and effect of osmotic forces on fluid exchange and ICP for the entire brain in both normal and pathological states (45). The secondary aim was to predict the effects of osmotic therapy for different doses in comparison with experimental data.

## METHODS

**Figure 2:**
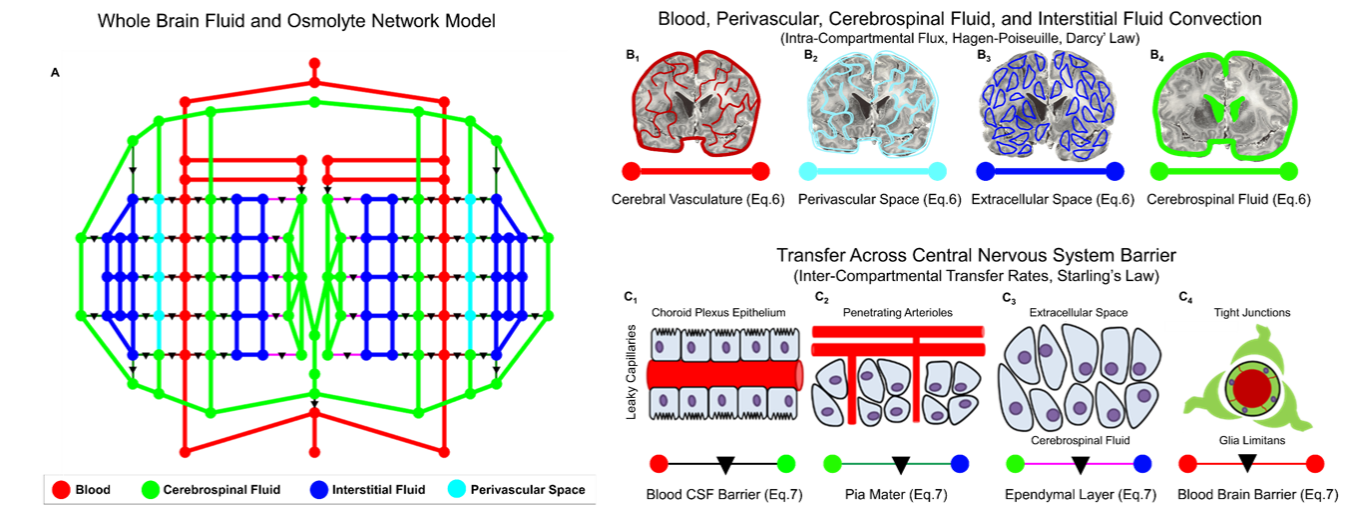
Mechanistic global network of the brain encompassing blood, perivascular space, cerebrospinal fluid, and tissue extracellular space. **A**. Network representation of the cerebral vasculature, perivascular spaces (PVS), cerebrospinal fluid (CSF) and interstitial fluid (ISF) flows throughout the brain. Circles (nodes, •) represent cerebral compartments. Red constitutes the vascular network. Light blue are the PVS. Blue is tissue, green marks CSF-filled spaces. **B**. Intra-compartmental arcs (•─•) connecting the nodes (blood, CSF, ISF) represent pathways for fluid and solute exchange. Blood, PVS, CSF, and ISF convection (inter-compartmental fluxes) obeys to Hagen-Pouiselle and Darcy’s law as shown in Frame B_1_-B_4_. (**C**) Inter-compartmental arcs transfer of water and osmolytes (solutes, mannitol or sucrose) is represented by triangulated arcs (•─▼─•) as shown in C_1_-C_4_ and include transfer across the BBB, the pial membrane, ependymal layers in pial-ventricular interfaces (CSF-tissue barrier) and blood-CSF barrier.

A global network was used to model fluid exchange throughout the brain. Connectivity was simplified into two structural elements: nodes and arcs. Nodes represented the main cerebral compartments, whereas arcs stood for types of fluid and solute exchange (Figure 2). The distributed network model had a total of 120 compartments, 150 single phase flows, and 61 transmembrane fluid and solute exchange fluxes (Table 1). The cerebral compartments were grouped into four subnets as follows: the cerebral circulation, the CSF system, the PVS, and ISF inside the brain parenchyma (Figure 2). The cerebrovascular subnet consists of arteries, arterioles, capillaries, venules, and veins. The interstitial space of the parenchyma is composed of ECS encompassing white matter and gray matter. The CSF system covers the lateral, third and fourth ventricle, the cisterna magna, and the cranial SAS. We also incorporated PVS, ie, the space between blood vessels and the parenchyma. Each node had four unknown states: hydrostatic pressure (P), osmotic pressure (Π), osmolyte concentration (C), and volume (V). The values of the unknowns would be determined by solving a mechanistic model of water exchange.

**Table 1:** Components and dimensions of model

Model Segment	Symbol	Number of Segments
Compartments	Node	120
blood	red	26
cerebrospinal fluid (CSF)	green	42
interstitial fluid (ISF)	blue	42
perivascular space (PVS)	light-blue	10
Single Phase Flow	Arcs	150
Barriers	Bracketed Arcs	61
blood-brain barrier (BBB)	black	10
blood-CSF	light-red	13
pia mater	dark-green	18
ependymal layer	cyan	20

Hydrostatic pressure differences determined flux between interior nodes of the blood, CSF, and ISF subnets. This type of arc was represented by straight lines connecting nodes of the same compartment. Blood and CSF flow obeys to Hagen-Poiseuille law, and ISF flux is given by Darcy’s law. In addition, solutes can be transported by convection in blood and CSF and by convection and diffusion in the tissue.

Fluid exchange between arterial compartments occurs via barrier membranes. Triangular-marked arcs connecting two different subnets represented the BBB, the blood-CSF, the ISF-CSF barriers in the periventricular spaces, the pial membrane, and the arachnoid villi. Transmembrane water exchange is governed by hydrostatic and osmotic driving forces known as Starling’s law. Solute transfer can be driven by transmembrane concentration differences or may be subject to active transport mechanisms. Water exchange occurs along arcs representing the BBB and the glia limitans connecting the cerebral circulation with the ECS. CSF production occurs by active processes, which create efflux from the choroidal capillaries into the ventricles. In addition, there is also water exchange between capillaries, PVS and ISF. Interstitial fluid in the ECS can enter CSF spaces via convection through the PVS or diffusion through the tissue.

At every node, water accumulation was tracked by the volume balance (Equation 1). Volume accumulation in each compartment was represented by 
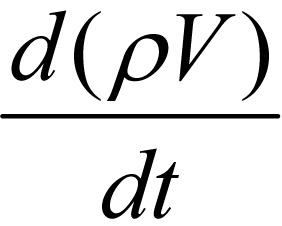
. Bulk flow of blood (water) was given as *Q*, and *J* represented transmembrane water fluxes. The divergence of the fluxes denoted by 
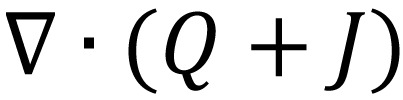
 was computed as a sum over all arcs entering each node.


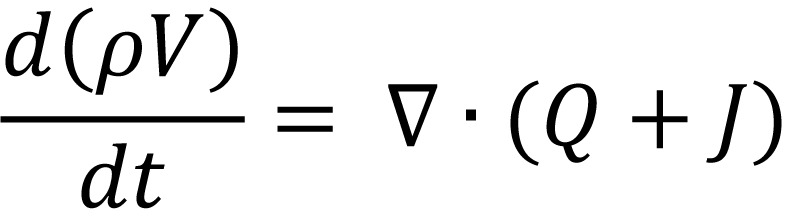
 Equation 1

Radiolabeled water is convected with concentration, *C_tcr_*, throughout cerebral blood plasma, PVS, ventricles, SAS, and the ECS. It can also diffuse across the membranes of the BBB, the pia mater, choroid plexus, and ependyma of the ventricles. Tracer concentration is determined by molar conservation balances (Equation 2). In the simple network described above, the concentration gradient, 
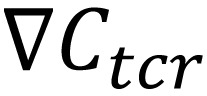
, was assumed to be equal to the difference of the concentration along a network arc, but could also be spatially discretized using orthogonal collocation or spectral differentiation methods (40,46).


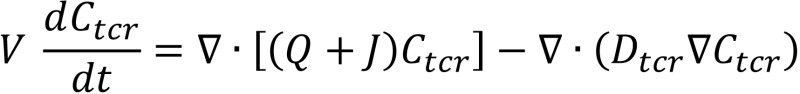
 Equation 2

In our simplified model, we only computed deviations from baseline osmolarity caused by a specific stimulus, such as the injection of an osmolyte (mannitol or sucrose). Therefore, changes to the osmotic pressure due to a stimulus could be computed as a departure from baseline osmolarity, 
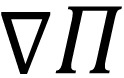
 (Equation 3). The osmotic pressure of a solution was approximated by the Van’t Hoff equation (Equation 4), where the osmotic pressure, *Π*, is related to the absolute temperature, T, the ideal gas constant, R, number of particles into which the molecule dissociates, *n*, and the molar concentration, *C_osm_*. The osmotic coefficient, φ, accounts for the degree of non-ideal solute dissociation. Here it was set to unity for both mannitol and sucrose.


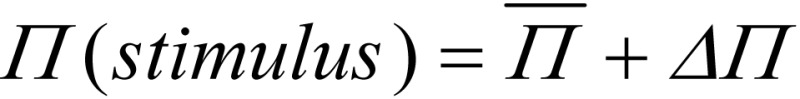
 Equation 3


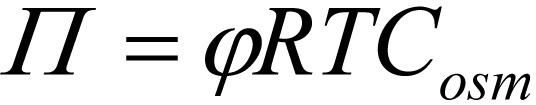
 Equation 4

The osmolyte concentration in each compartment, *C_osm_*, obeys to the species conservation balances, ie, osmolyte transport in blood and CSF follows Equation 5 and osmolyte transport in tissue follows Equation 6. Osmolytes, ie, sucrose and mannitol, are also transported due to convection inside the vascular and CSF compartments. In tissue, osmolytes diffuse according to the diffusion coefficient, *D_osm_*. Osmolytes can cross semi-permeable membranes driven by concentration gradients between the two compartments, *C_1_* and *C_2_*. In a first approximation, osmolyte flux across the BBB is governed by the surface area between the compartment, *S*, and the mass transport coefficient, *U*. Different interfaces, such as vasculature-ventricle, tissue-ventricle, tissue-SAS (*i*), possess different mass transfer coefficient and surface areas according to physiological differences (Supplementary material 3 (supplementary material 3)).


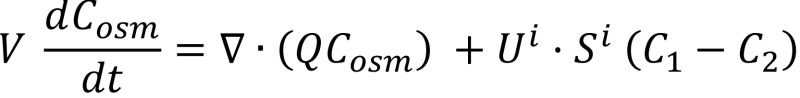
 Equation 5


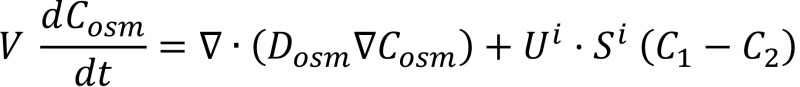
 Equation 6

Blood flow obeys the Hagen Poiseuille equation (Equation 7). Pressure-driven convection also governs CSF flow in ventricles and the SAS. The volumetric bulk flow rate of water, *Q*, depends on the pressure drop across the vessel, 
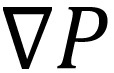
, and the hydraulic resistance of the vessel, *α*. Resistances are assigned by representing the vascular and CSF compartments as idealized cylinders with equivalent hydrodynamic lengths, *L*, and radii, *r*. The computed resistance is also a function of the dynamic fluid viscosity, *μ*. Bulk blood flow occurs through arteries, arterioles, capillaries, veins, and venules, ISF flows in the ECS and PVS, and CSF travels from the ventricles to the cranial SAS.


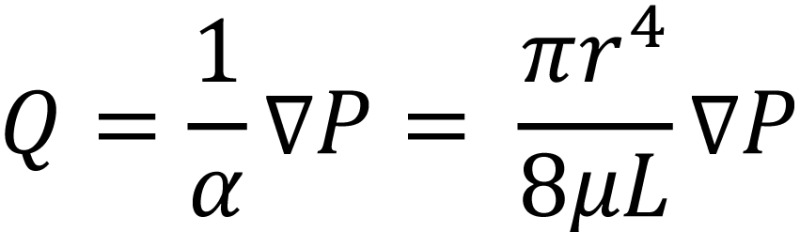
 Equation 7

Water exchange occurs across the membranes of the choroid plexus, BBB, ECS, the pia matter, and ependymal layer lining the ventricles. Water transfer across membranes is governed by Starling’s Law (Equation 8). Here the transmembrane water flux, *J*, depends on the hydraulic conductivity, *L_p_*, the reflection coefficient of the solute, σ, and the osmotic pressure gradient, 
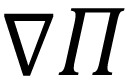
. Both the bulk, *Q,* and transmembrane water flux, *J,* contribute to water exchange in the brain. Only the transmembrane fluid exchange, *J,* is a function of both hydrostatic and osmotic pressure gradients. Bulk flows, *Q*, depends only on hydrostatic pressure differences.


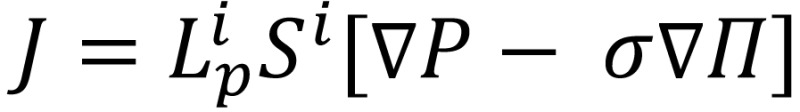
 Equation 8

ECS water transport is approximated by Darcy’s law describing flux in a porous media (Equation 9). The bulk flow, Q_ECS_, is computed from the hydrostatic pressure gradient, 
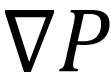
, cross-sectional area of the interface between the capillaries and the ECS, *A_Cap-ECS_*, the ECS permeability, *k*, for water and the hydraulic length of the porous compartment, *L.*


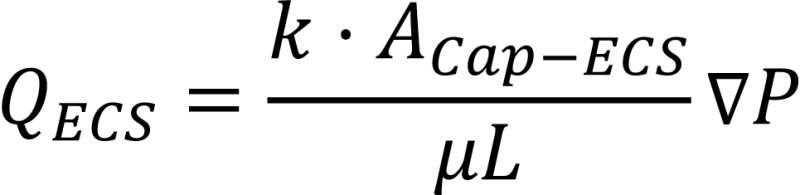
 Equation 9

The compliance of each compartment is approximated by relating volume change, 
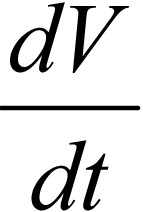
 at each node to the compliance, *Γ(V),* and the ICP change, 
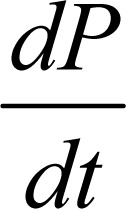
 (Equation 10). Physiologically, the pressure-volume relationship within the cranial vault is governed by the Monro-Kellie doctrine. A nonlinear compliance term accounts for physiological pressure-volume relationship (47-49).


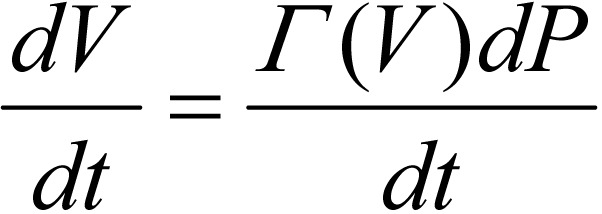
 Equation 10

The system of nonlinear differential equations for hydrostatic and osmotic pressures, osmolytes, and volume was solved iteratively in MATLAB by implicit time integration. A pressure drop of 100 mmHg between the most distal arterial and venous nodes was enforced. Intravenous or intra-ventricular administration of an osmolyte was introduced and caused a shift in local osmolarity. The transient and steady state changes in water exchange, *J*, between cerebral compartments were predicted using a finite volume method enforcing balance equations (Supplementary material 3 (supplementary material 3)).

## RESULTS

### Prediction of the effect of ventriculo-cisternal perfusion on intracranial pressure

**Figure 3:**
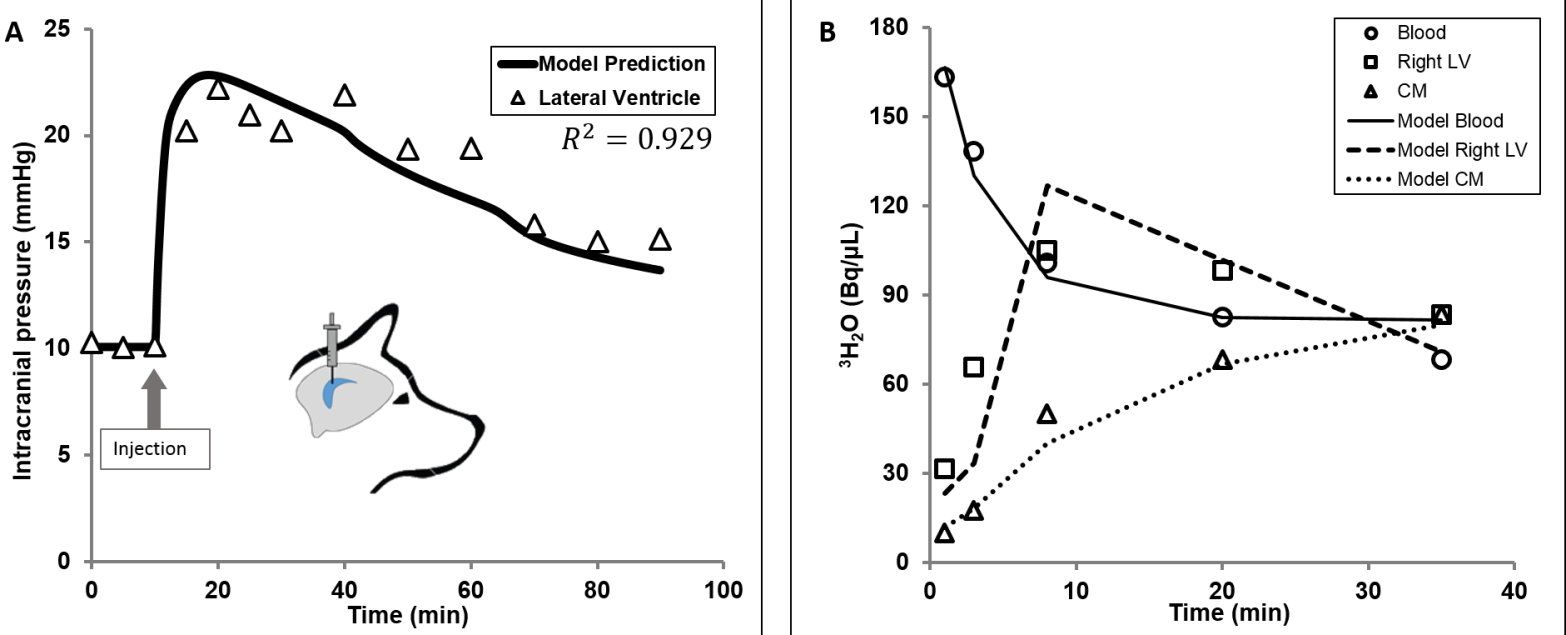
Computational predictions of intracranial pressure (ICP) rise following hyperosmolar sucrose infusion to the lateral ventricle is compared against experimental data. **A.** The model prediction is in good agreement with the ICP rise measured in vivo by Klarica et al (18), R^2^ = 0.929, following hyperosmolar sucrose infusion in the lateral ventricle. **B.** Radiolabeled water in the systemic circulation is tracked following the injection of hyperosmotic sucrose. The comparison between the predictions of the model and the experimental data show that the model is capable of reproducing the trends of both the effect of osmotic pressure gradients on intracranial pressure and water exchange between the blood and cerebrospinal fluid.

As the movement of radiolabeled water ^3^H_2_0 may be traced in anaesthetized adult mongrel dogs (18), a bolus of 4 mL radiolabeled water was injected into the femoral vein. Simultaneously a 1 mL hyperosmolar sucrose solution with a concentration of 950 mmol/L was injected into the lateral ventricle to raise CSF osmolarity. The observed CSF pressure rise was measured via a catheter inserted into the right lateral ventricle. Model predictions and the experimental data were compared in Figure 3. Concurrently, the ventricular volume also increased significantly by a factor of 3-4 (data not shown).

The predicted increase in CSF pressure after hyperosmotic sucrose injection matched the experimental data with a coefficient of determination R^2^ = 0.929 (Figure 3A). Baseline and maximum CSF pressures before and after injection of hyperosmotic sucrose into the lateral ventricle predicted by the model were 14.2 and 31.4 mmHg and agreed with the experimental results of 14.0 and 30.4 mmHg, with only 1.4% and 3.2% error, respectively. Moreover, simulated dynamic tracer distribution, C_tcr_, agreed in timing and magnitude of measured plasma, right lateral ventricle, and the cisterna magna, with R^2^ values of 0.957, 0.899, and 0.981, respectively (Figure 3B).

### Simulation of osmotic therapy for clearance of cerebral edema

**Figure 4:**
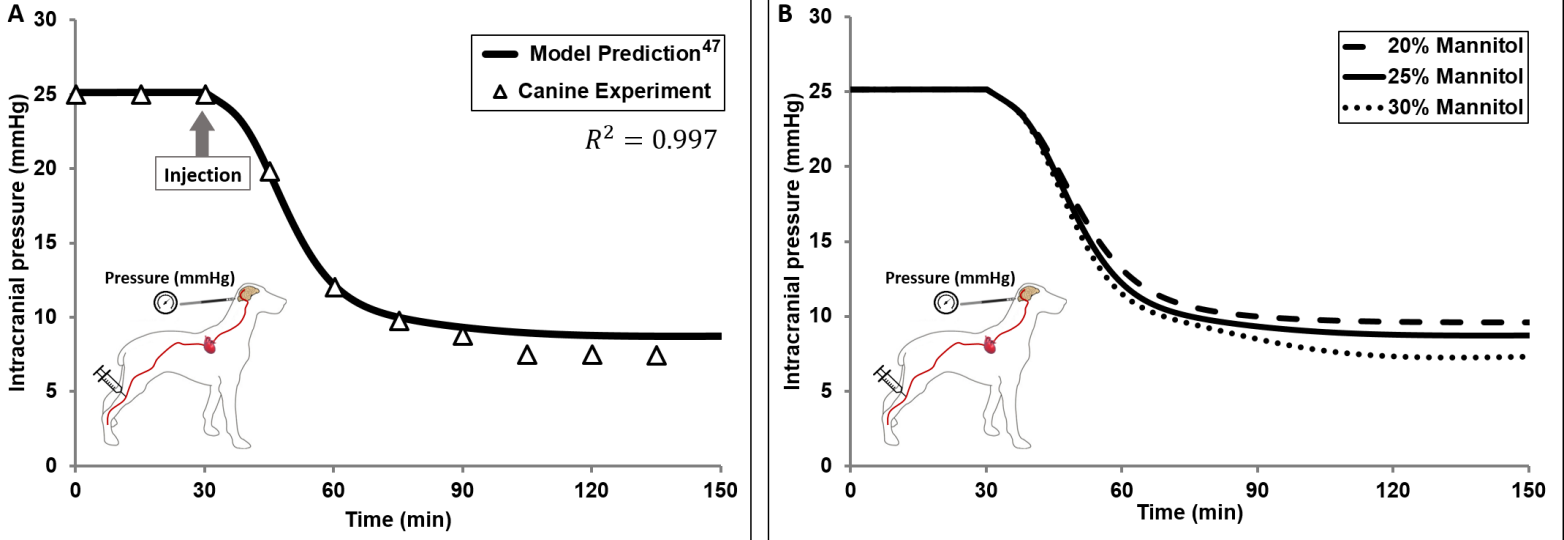
Simulated effect of osmotic therapy on mediating edema induced intracranial pressure (ICP) increase and the resulting recovery following an intravenous injection of hyperosmotic mannitol solution. **A**. Model predictions match the experimental ICP trajectory following osmotic therapy, conducted by Israel et al (50). **B**. Simulated ICP trajectories for different concentrations of mannitol solutions quantify the degree of ICP reduction achievable by systemic osmolarity increase. Higher concentrations of administered mannitol doses are only slightly predicted to accelerate the speed of the ICP decrease.

Increased ICP after edema is clinically managed by intravenous injection of hyperosmotic mannitol solution, which decreases ICP over the course of 2.5 hours, a procedure known as osmotic therapy (Figure 4). Israel (50) simulated the effect of edema on ICP, by inserting a balloon in the lateral ventricle of a dog (Figure 4A). This procedure caused a 15 mmHg pressure increase, which was managed by osmotic therapy. IV injection of mannitol lowered ICP from 25 mmHg to 8 mmHg. The simulation of the osmotic therapy matched the time course and magnitude of the ICP recovery of the experimentally measured data, with an R^2^ = 0.997. Further reduction of ICP could be achieved with three different doses of mannitol (Figure 4B).

### The effect of perivascular space on tracer dispersion

**Figure 5:**
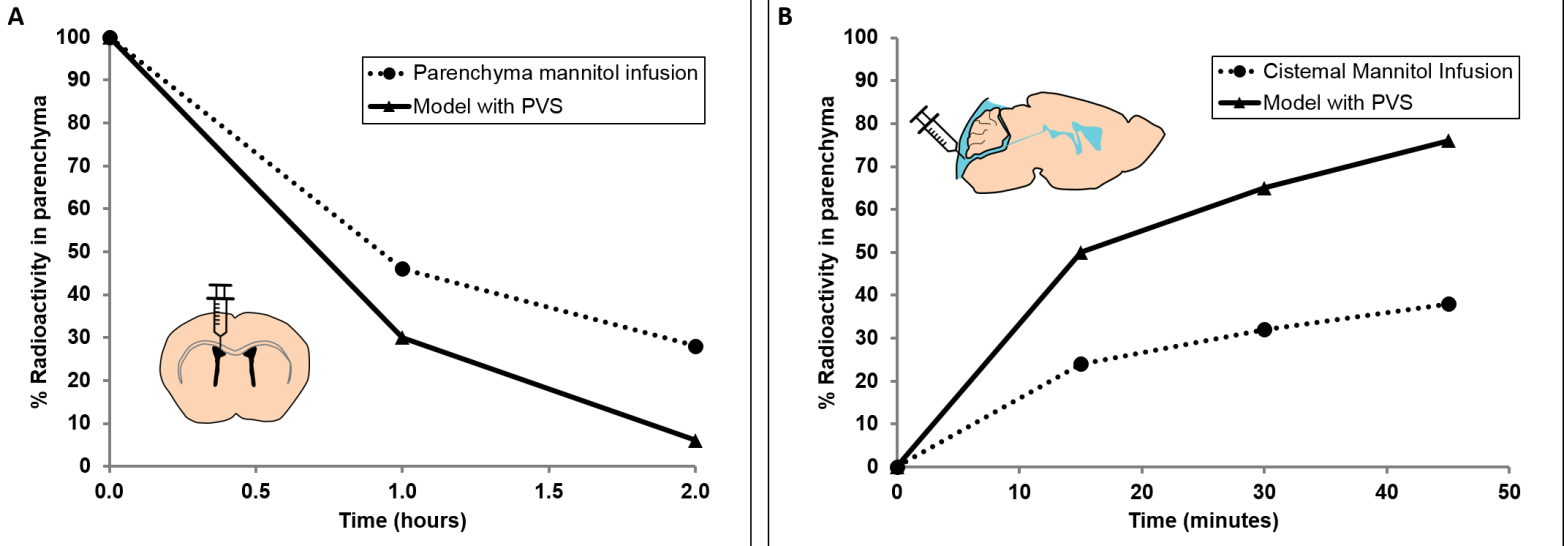
Comparison between experimental and computationally predicted trajectories of radiolabeled mannitol flux between the parenchyma and the cerebrospinal fluid (CSF). **A**. Simulated predictions of clearance of radiolabelled mannitol out of the parenchyma shows good agreement with the trends seen *in vivo* experiments conducted by Iliff et al (2). **B.** The trend of movement of radiolabeled tracer into the parenchyma after injection into the cisterna magna is similar to model predictions. However, the model overestimates the magnitude and rate of mannitol flux. The presence of the perivascular space, which acts as a conduit between the subarachnoid CSF and extracellular space of the brain, significantly increases the speed at which tracer is distributed between these compartments.

Iliff et al (2) used radiolabeled mannitol to quantify the speed of solute exchange between the cisternal CSF and the parenchyma. The study measured both solute influx from the cisterna magna into the brain tissue and the rate of solute clearance from the parenchyma. Model predictions were qualitatively compared with experiments, with both sources showing the clearance of radiolabeled solute (Figure 5). Specifically, the model predicted that after 2 hours, more than 90% of the radiolabeled mannitol had been removed from the parenchyma, whereas the experimental data suggested about 70%. After 45 minutes, the model predicted that nearly 75% of the mannitol injected into the cisterna magna had reached the parenchyma. The experimental data showed a slower influx of merely 40% into the parenchyma. The current model overestimates mannitol clearance from the parenchyma into the CSF (Supplementary material 2 (supplementary material 2)).

### Simulation of CSF production and capillary reabsorption

*Intravenous osmolyte infusion*. The model was used to reproduce classical ventriculo-cisternal perfusion experiments by Dimattio et al (15), who measured the effect of osmotic pressure gradients between the blood and CSF on nascent fluid production rate. Dog experiments showed that nascent fluid flow rate of 22.7 µL/min remained unaltered during isomolar intravenous control injection of 322 mOsm (control state with no change in production). However, a 360-mOsm injection (hyperosmolar) essentially inhibited nascent fluid flow. In contrast, hypo-osmolar infusion (290 mOsm) enhanced CSF production by 220%. Osmolarity challenges introduced in ventricles achieved the opposite effect on nascent fluid generation with a slope of -0.79 mOsm/µL/min. The slope predicted by the model amounted to -0.5 mOsm/µL/min (Supplementary material 2 (supplementary material 2)). Experimental and predicted results are also provided in Supplementary material 2. (supplementary material 2)

*Intraventricular osmolyte infusion*. Wald et al (14) controlled the CSF osmolarity of the lateral ventricle in a canine model by sucrose infusion within a range of 6-780 mOsm. Isotonic sucrose solution (320 mOsm) yielded a 24.9 μL/min nascent fluid flow rate as baseline. Injection of a 6 mOsm solution (hypo-osmolar) completely stopped nascent fluid flow. Hyperosmolar environment (780 mOsm infusion) increased the production rate by more than 350%. Linear regression of the nascent fluid production rate due ventricular osmolarity change was 0.25 mOsm/µL/min (Supplementary material 2(supplementary material 2)). The model gave a slope of 0.15 mOsm/µL/min, but could not reproduce the progressive fluid generation increase at high osmolarity.

## DISCUSSION

A first mathematical model for water exchange due to Starling forces was developed with a global scope for cerebral circulation, brain ISF, cranial CSF, and the PVS. It accounted for the contributions of classical Starling forces due to osmolytes on cerebral water shifts and solute exchange across the BBB. It is critical for the model to not merely predict known trends in isolation, but rather to introduce a spatially distributed, anatomically consistent, mechanistic network model of the intracranial dynamics for the entire brain. Results quantitatively agree with experimental data measuring the effects of osmotic pressure gradients on intracranial water flux and ICP. The model was able to reproduce the experimentally observed dynamic trends of radiolabeled water distribution between the blood, lateral ventricles, and cisterna magna observed in experiments. It also described the alteration in nascent fluid production rates in response to osmotic gradients between blood and CSF. Furthermore, the model corroborated and quantified the possibility to control ICP by osmotic forces. The results further suggest that our hypothesis quantifying the relationship between water flux, pressure, and osmolyte concentration was successful in reproducing the large body of experimental evidence including ventriculo-cisternal perfusion injections and osmotic therapy.

Moreover, the model underscored a possible role of the PVS in ISF exchange. It was able to quantify the increased speed of both mannitol clearance from the parenchyma and penetration from the SAS into the parenchyma as observed in recent tracer infusion experiments (2).

The proposed research on water transport for the brain has potential clinical implications in the future. For example, it could inform about the optimal timing and dosing of osmotic therapy. This is significant because under-dosing may fail to lower the highly dangerous, acute ICP elevations that occur during cerebral edema. On the other hand, overdosing can potentially harm osmotically sensitive organs, such as the liver and kidney, and should be avoided.

However, limitations of the computational model are rooted in the complex nature of cerebral transport phenomena. Regulation of cranial water flux employs a multitude of mechanisms, both mechanical and biochemical, that are not yet accounted for in the current model. For example, vasoconstriction for blood flow control or mediation of disease states, such as cerebral edema, are not incorporated. Furthermore, while the model is able to quantify filtration and reabsorption phenomena across the capillary endothelium (hydrostatic and osmotic pressure), there are additional mechanisms for the transport of water across the membranes separating the intracranial compartments. It has been shown that a significant amount of water transport between the blood and the ECS may be due to transcellular co-transport with ions and glucose through membrane-bound protein channels (51-53).

Additionally, revised Starling’s Law was not yet incorporated into the computational model (Supplementary material 4(supplementary material 4)). The revised Starling’s Law would reduce capillary filtration rate at low concentrations (54). Furthermore, the morphology and the dynamics of perivascular and extracellular space modulation are subjects of active ongoing research activity and require more data (2,22,24,31). As more quantitative data on perivascular transport become available, future revisions could be informed by these insights and more precise measurements. We also did not attempt to address molecular aspects of extravascular transport, although much progress in this direction has been made recently (31).

The whole brain network approach represents a first step in construction of a mechanistic model incorporating the major cranial compartments and key phenomena to describe the physiochemical interaction between water and solutes, intracranial pressures, and compartmental volumes in the brain.

Despite its critical significance for blood supply and normal brain function, mechanistic factors determining ICP are poorly understood. Our theoretical model based on *in vivo* experiments suggests that ICP is tightly linked to physical (hydrostatic) and chemical (osmotic) driving forces. Our computational results suggest that critical ICP elevations >30 mmHg, which may cause insufficient cerebral perfusion, may be treatable by pharmacological methods. Specifically, suitable administration of osmotic agents may aid in establishing osmotic gradients between cerebral compartments that drain excess fluid responsible for dangerous brain swelling. Quantitative knowledge about water exchange fluxes between blood, extravascular spaces (interstitial fluid), and the CSF will help establish the direction and extent of achievable ICP control with pharmacological means. Thus, rational design of osmotic therapy has implications in treating brain edema and traumatic brain injury.

Chronic imbalance of water content is a symptom in hydrocephalus. Enlarged ventricles, the characteristic indication for hydrocephalic conditions, are still commonly associated with CSF overproduction. This belief is clearly inaccurate, because even in a brain with enlarged ventricles, CSF production is matched by commensurate removal as follows from simple mass conservation. Instead, ventriculomegaly often accompanied by ICP increase indicates a shift in water exchange fluxes among cerebral compartments. Specifically, ventricular enlargement has been implicated with reduced extracellular spaces. The physicochemical theory of the interconnected cerebral water exchange suggests that induction and maintenance of chronic size shifts is likely to involve chemical (osmotic) factors. A mechanistic understanding of pathological water shifts may open the door for non-invasive treatment of hydrocephalus. Instead of draining CSF by mechanical shunting, which is prone to revision due to valve failure, we may discharge excess fluid from the ventricular system by a molecular shunt. In a future molecular shunt system, pharmacologically established osmotic gradients between the vascular bed and astrocytes could open the existing aquaporin channels capable of siphoning excess water content by natural physiological means instead of surgically placed shunt valves. The presented theory aims to lay a theoretical foundation informing research in this innovative direction.
