# Supplementary material for: Starling forces drive intracranial water exchange during normal and pathological states
Source: Croat Med J. 2017 Dec;58(6):384–94. doi: 10.3325/cmj.2017.58.384 (PMC5778682; doi:10.3325/cmj.2017.58.384)
Supplement: Supplementary Material 1 [file CroatMedJ_58_s001.pdf]

Supplementary material 1. Evolution of CSF production hypothesis from classical unidirectional flow to the Bulat-Klarica-Orešković hypothesis

Author	Study Type	Main Finding
Weed (1914)	In vivo study w/ dogs, cats, and monkeys	The majority of CSF is returned to the circulation via arachnoid villi with accessory drainage of CSF into lymphatic.
Dandy (1919)	In vivo study w/ dog (n=1)	CSF is formed from the CP, and the ependyma does not secrete CSF.
Cushing (1925)	Lecture summarizing pre-1920 findings	Unidirectional CSF flow was described as the "third circulation".
Milhorat (1969)	In vivo study w/ rhesus monkeys (n=149)	Hydrocephalus occurred in monkeys with ventricular obstruction even when the CP was removed. Furthermore, CSF composition after CP removal was unchanged.
Milhorat, *et al.* (1971)	In vivo study w/ rhesus monkeys (n=17)	Choroid plexectomy reduced the production of CSF by an average of 33-40%. Therefore, the CP is not the sole source of CSF production.
DiMattio, *et al.* (1975)	In vivo study w/ cats (n=17)	Decreasing white and grey matter water content coincided with decreases in ventricular bulk flow when concentrated glucose was infused into the blood stream.
Wald, *et al.* (1976)	In vivo study w/ cats (n=60)	Increased bulk flow induced through hypertonic solution perfusion originates from the CP.
Wald, *et al.* (1977)	In vivo study w/ cats (n=16)	Decreasing serum osmolarity resulted in CSF volume flow increasing and tracers initially in the white matter to appear in the ventricular system.
Upton, *et al.* (1985)	Human brain microscopy (n=23)	Erythrocytes were found in arachnoid granulations after subarachnoid hemorrhage suggesting the AG are connected to the SAS and are CSF drainage pathways.
Pople, *et al.* (1995)	Retrospective review of human patients (n=104)	CP coagulation is not effective for treating hydrocephalus as 65% of patients required a catheter for long-term hydrocephalus control. Ventricular size was not impacted.
Kapoor, *et al.* (2008)	Review paper	Multiple pathways and methods of CSF drainage were reviewed.
Bulat, *et al.* (2008)	In vivo study w/ cats (n=4)	CSF volume does not flow unidirectionally along CSF spaces, instead water (99% of CSF volume) is absorbed transventricallly into periventricular capillaries.
Klarica, *et al* (2009)	In vivo study w/ cats (n=10)	Blockage of aqueduct of Sylvius does not increase CSF pressure or induce ventricular dilation.
Maraković, *et al. (2010)*	In vivo study w/ cats (n=12)	Perfusion of CSF with hyperosmolar CSF leads to higher outflow volume during ventriculo-cisternal perfusion compared to perfusion with iso-osmolar CSF.
Maraković, *et al. (2012)*	In vivo study w/ cats (n=4)	Application of distilled water increases CSF outflow volume and CSF pressure.
Klarica, *et al.* (2013)	In vivo study w/ cats (n=6) and dogs (n=23)	Sub-chronic application of hyperosmolar solution to ventricles results in hydrocephalus development without any obstruction of CSF pathways.
Yamada (2014)	Human imaging: time-SLIP MRI	CSF was found to exhibit pulsatile movement, but no CSF circulation from site of production to site of drainage.
Matsumae, *et al.* (2014)	Human Imaging: 3D-PC MRI (n=13)	Velocity field from 3D-PC MRI was converted to pressure gradient. CSF motion was stagnant at the CP yielding a minimal pressure gradient suggesting the CP is not a pump.
Orešković, *et al.* (2017)	Review paper	The Bulat-Klarica-Orešković hypothesis posits that CSF exchange is present everywhere in the CSF system and is a consequence of water filtration between capillaries and ISF.
