# Supplementary material for: Starling forces drive intracranial water exchange during normal and pathological states
Source: Croat Med J. 2017 Dec;58(6):384–94. doi: 10.3325/cmj.2017.58.384 (PMC5778682; doi:10.3325/cmj.2017.58.384)

Supplementary material 2. Osmolarity influence on nascent fluid production

Osmotic pressure gradients and nascent fluid production

The extent of cerebrospinal fluid (CSF) production and reabsorption has been linked with the osmolarity of blood and CSF (14-19) suggesting that the normal balance of osmotic pressures between the cerebral blood plasma, extracellular space, and CSF determines the extent of water and solute exchange between these compartments (water homeostasis). The body of evidence derived from ventriculo-cisternal perfusion (VCP) experiments (Supplementary figure 1) shows that artificially induced osmolarity changes in the lateral ventricles (12,13) significantly affect CSF production. The degree of tracer dilution collected in fluid from the cisterna magna can be used to determine the bulk flow rate of fluid generated inside the ventricle; this quantity is termed *nascent fluid*. It should be noted that these experiments quantify, but do not elucidate the mechanism of fluid production, which may be due to active CSF secretion, filtration across the choroid plexus epithelium, or filtration across the ependymal layer of the ventricles.

Nascent fluid production has experimentally been altered by changing plasma osmolarity (12,15,16), as summarized in Supplementary figure 1. Jurjević et al (15) increased serum osmolarity by hyperosmolar intravenous injection of mannitol. Hyperosmotic serum has been shown to severely reduce and even completely inhibit nascent fluid formation, while hypo-osmotic serum has been shown to double it. Wald et al (12) also measured the effect of CSF osmolarity changes in the ventricles on nascent fluid production. CSF osmolarity in the ventricle was determined to enhance bulk fluid production by 0.231 μL per mOsm. Klarica et al (16) traced the exchange of radiolabeled water, $^3H_20$, across the blood-brain barrier and quantified the effect of hyperosmolar injection into the lateral ventricle of a dog by measuring the radioactivity in fluid collected from the cisterna magna. The rate of radiolabeled tracer accumulation increased significantly when compared to isomolar controls.

These studies demonstrate that osmotic pressure gradients are a significant driving force not only for CSF production, but also for water filtration and reabsorption across the blood brain barrier, blood-CSF barrier (choroid plexus epithelium), as well as the CSF-ISF barrier (ependymal layer of the ventricles). These physiochemical transport phenomenon have so far not been incorporated into CSF and fluid exchange models of the brain.

Supplementary figure 1. Summary of classical and recent ventriculo-cisternal perfusion experiments that measure, as a function of serum or CSF osmolarity, the production rate of nascent fluid. Schematic illustrating ventriculo-cisternal perfusion experiments by Klarica et al (16). Nascent fluid production was inferred indirectly by measuring the dilution of radiolabeled tracer in samples taken from the cisterna magna. Nascent fluid production is reduced with increasing serum tonicity or decreasing CSF tonicity. Conversely, nascent fluid production can be augmented by hypo-osmolar serum tonicity or hyperosmotic injection into the CSF spaces.

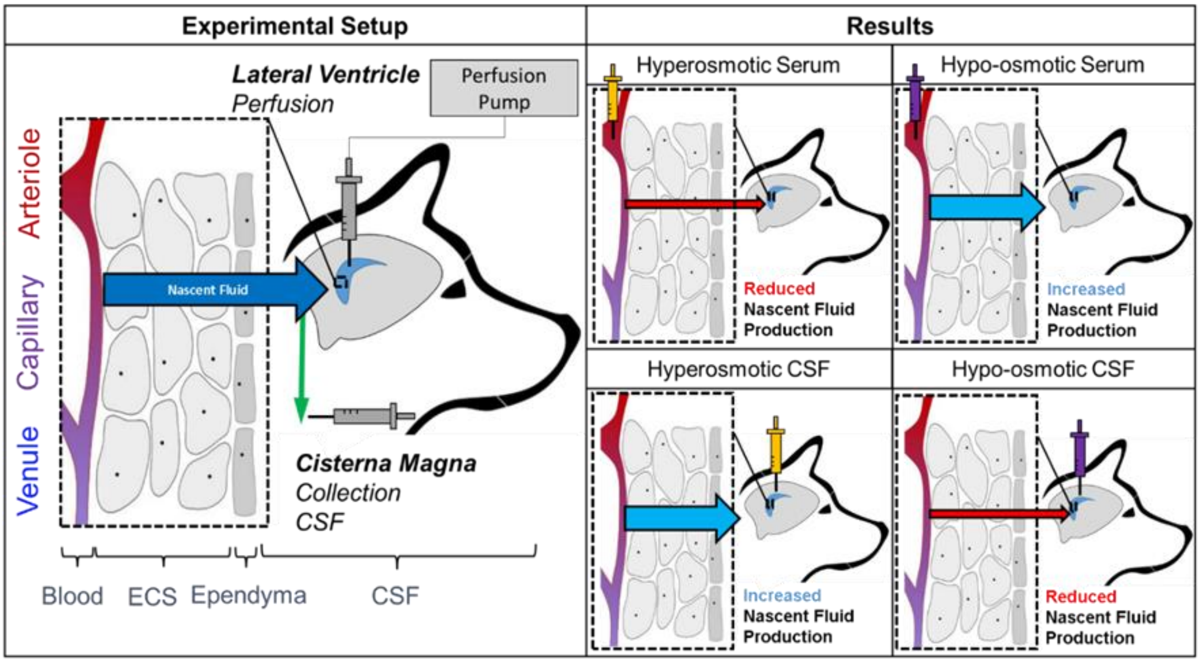

Simulation of nascent fluid production in response to osmolar challenge. The computational model was also used to predict nascent CSF production as a function of osmolarity. Two scenarios were evaluated to compare to experimental studies in the literature. Intravenous osmolyte infusion conducted by Dimattio et al (13), with results in Supplementary figure 2A. Complimentary experiments with alterations of CSF osmolarity by Wald et al (12) are summarized in Supplementary figure 2B. For both cases, simulations follow the experimental trends qualitatively. The progressive effect of hyper-osmolar CSF on nascent fluid production could not be reproduced in the model. We conclude that the classical Starling's law is insufficient to capture compounding non-linear effects in the high osmolarity range.

Supplementary table 2. Summary of experimental values for bulk flow of nascent fluid due to osmotic challenge of the serum or the ventricle measured via ventriculo-cisternal perfusion, compared to model predictions

	**Injection, mOsm**	**Model Bulk Flow, µL/min**	**Experimental Bulk Flow, µL/min**
**Ventricle**	6	5.2	0.0
	320	20.6	24.9
	780	75.2	88.0
**Serum**	290	40.4	49.9
	322	19.5	22.7
	360	4.6	0.0

Ventricular sucrose infusion. Wald et al controlled the CSF osmolarity of the lateral ventricle in a canine model by sucrose infusion within a range of 6-780 mOsm. Isotonic sucrose solution (320 mOsm) yielded a 24.9 µL/min nascent fluid flow rate as baseline. Injection of a 6 mOsm solution (hypo-osmolar) completely stopped nascent fluid flow. Hyperosmolar environment (780 mOsm infusion) increased the production rate by more than 350%. Linear regression of the nascent fluid production rate due ventricular osmolarity change was 0.25 mOsm/µL/min (Supplementary figure 2). The model gave a slope of 0.15 mOsm/µL/min and could not reproduce the progressive fluid generation increase at high osmolarities.

Supplementary figure 2. Classical VCP experiments demonstrating that osmolarity changes in the serum (**A**) or lateral ventricle infusion (**B**) alter nascent fluid production. The computational model shown as a solid line replicates the trends of experimental data. Serum osmolarity was inversely correlated with nascent fluid production. Osmolarity changes induced by CSF infusion were linearly correlated with nascent fluid production. The model was able to predict the trends of increase and decrease of nascent fluid production over a wide range of osmotic pressures ranges, except for deviations at high osmolarity ranges.

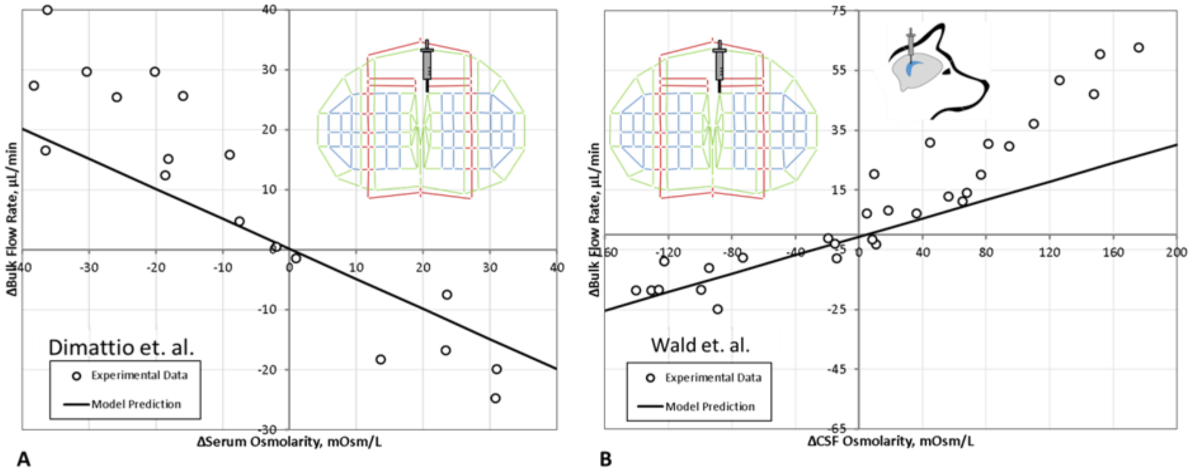

Supplement: Supplementary Material 2 [file CroatMedJ_58_s002.pdf]
