# Supplementary material for: Starling forces drive intracranial water exchange during normal and pathological states
Source: Croat Med J. 2017 Dec;58(6):384–94. doi: 10.3325/cmj.2017.58.384 (PMC5778682; doi:10.3325/cmj.2017.58.384)
Supplement: Supplementary Material 3 [file CroatMedJ_58_s003.pdf]

**Supplementary material 3.** Model parameters for the mechanistic brain model simulations of osmotic challenge to the blood or ventricular CSF are included in Table A1.

**Supplementary table 3.** Parameters defining the computational model

| Property | Symbol | Value | Unit |
|---|---|---|---|
| **Physical Properties of Dog Brain** | | | |
| **Property** | **Symbol** | **Value** | **Unit** |
| Parenchyma Surface Area | $S_p$ | 1400 | $mm^2$ |
| Lateral Ventricle Surface Area | $S_v$ | 40 | $mm^2$ |
| Brain Volume | $V_{br}$ | 47 | $mL$ |
| Lateral Ventricle Volume | $V_v$ | 1 | $mL$ |
| Blood Volume | $V_{bl}$ | 5 | $mL$ |
| Subarachnoid Space Volume | $V_{sas}$ | 1 | $mL$ |
| **State Properties of Model** | | | |
| **Property** | **Symbol** | **Value** | **Unit** |
| Arterial-Venous Pressure Drop | $\Delta P$ | 100 | $mmHg$ |
| Arterial Blood Pressure | $P_A$ | 23-105 | $mmHg$ |
| Capillary Blood Pressure | $P_{Cap}$ | 15-23 | $mmHg$ |
| Venous Blood Pressure | $P_V$ | 5-15 | $mmHg$ |
| Temperature | $T$ | 310 | $K$ |
| Gas Constant | $R$ | 62.363 | $L \cdot mmHg \cdot K^{-1} \cdot mol^{-1}$ |
| **Transfer Coefficients** | | | |
| **Property** | **Symbol** | **Value** | **Unit** |
| Osmotic Dissociation Constant | $\varphi$ | 1.0 | -- |
| Oncotic Deflection Coefficient | $\sigma$ | 1.0 | -- |
| Solute Diffusivity | $D_{osm}$ | $5.75 \cdot 10^{-1}$ | $mm^2 \cdot s^{-1}$ |
| Tracer Diffusivity | $D_{trc}$ | N/A | $mm^2 \cdot s^{-1}$ |
| Vasculature-SAS Mass Transfer | $U_{vasc-SAS}$ | $1.12 \cdot 10^{-4}$ | $mm \cdot s^{-1}$ |
| Ventricle-Tissue Mass Transfer | $U_{vent-tissue}$ | $3.93 \cdot 10^{-3}$ | $mm \cdot s^{-1}$ |
| Tissue-SAS Mass Transfer | $U_{tissue-SAS}$ | $2.8 \cdot 10^{-5}$ | $mm \cdot s^{-1}$ |
| Vasculature-SAS Hydraulic Conductivity | $L_{p_{vasc-SAS}}$ | $3.37 \cdot 10^{-14}$ | $m^2 s \cdot kg^{-1}$ |
| Ventricle-Tissue Hydraulic Conductivity | $L_{p_{vent-tissue}}$ | $1.77 \cdot 10^{-4}$ | $m^2 s \cdot kg^{-1}$ |
| Tissue-SAS Hydraulic Conductivity | $L_{p_{tissue-SAS}}$ | $2.52 \cdot 10^{-7}$ | $m^2 s \cdot kg^{-1}$ |
| **Resistances** | | | |
| **Property** | **Symbol** | **Value** | **Unit** |
| Blood Vessel Radius | $r_{blood}$ | 2.5 | $mm$ |
| Blood Viscosity | $\mu_{blood}$ | 5.0 | $g \cdot mms^{-1}$ |
| Blood Vessel Length | $L_{blood}$ | 1.0 | $mm$ |
| CSF “Vessel” Radius | $r_{CSF}$ | 0.25 | $mm$ |
| CSF Viscosity | $\mu_{CSF}$ | 5.0 | $g \cdot mm \cdot s^{-1}$ |
| CSF “Vessel” Length | $L_{CSF}$ | 1.0 | $mm$ |
| Tissue Permeability | $\kappa_{tissue}$ | 1.0 | $mm^2 s \cdot g^{-1}$ |
| Tissue Surface Area | $A_{tissue}$ | $785 \cdot 10^{-6}$ | $mm^2$ |
| Tissue Length | $L_{tissue}$ | 1.0 | $mm$ |
| ISF Viscosity | $\mu_{ISF}$ | 5.0 | $g \cdot mm \cdot s^{-1}$ |
| PVS Permeability | $\kappa_{PVS}$ | 1.0 | $mm^2 s \cdot g^{-1}$ |
| PVS Surface Area | $A_{PVS}$ | $785 \cdot 10^{-6}$ | $mm^2$ |
| PVS Length | $L_{PVS}$ | 1.0 | $mm$ |
