# Supplementary material for: Starling forces drive intracranial water exchange during normal and pathological states
Source: Croat Med J. 2017 Dec;58(6):384–94. doi: 10.3325/cmj.2017.58.384 (PMC5778682; doi:10.3325/cmj.2017.58.384)
Supplement: Supplementary Material 4 [file CroatMedJ_58_s004.pdf]

Supplementary material 4. Comparison between Starling's and revised Starling's Law

Revised Starling's Law. The microcirculation hypothesis on cerebrospinal fluid (CSF) generation throughout the brain is not without theoretical basis. It was observed by Starling in 1896 that the change in osmolarity caused water transfer across tissue membrane barriers. The classical Starling Law states that differences between both the hydrostatic pressure, ∇P , and osmotic pressure gradients, $\nabla \Pi$ determine the extent of transmembrane flux, J . Accordingly, the classic Starling's Law in equation A1, predicts the production or absorption of water across the blood brain barrier.

Starling's Law | Equation

$$J = L_p S[\nabla P - \sigma \nabla \Pi] \qquad \text{A1}$$

Revised Starling's Law

$$\frac{J_V}{A} = L_p \left[\nabla P - \sigma^2 \cdot \Pi_p \cdot \frac{(1 - e^{-Pe})}{(1 - \sigma . e^{-Pe})} \right] \qquad \text{A2}$$

$$Pe = J_V \left(\frac{1 - \sigma}{P_d A} \right) \qquad \text{A3}$$

It has been accepted by many physiologists that the classic Starling Law predictions differ from experimental observations and over-estimated interstitial fluid osmotic pressures (26,27). This effect is accounted for in the revised Starling's Law (Equations A2 and A3), which couples solute and water transfer across the membrane. According to the revised Starling's Law, increasing the osmolarity drop across the endothelial- glycocalyx membrane causes a gradually diminishing efflux without the ability to reverse flow. Even though it is beyond the scope of this article to look at the revised Starling's Law, it is important to examine, based on pressure and osmolarity, in which direction and to what magnitude filtration and absorption can occur.

The directionality of transmembrane fluxes are compared for multiple scenarios of varying the pressure and osmolarity differences across the blood-brain barrier.
